# Omental infarction as an unusual manifestation of decompensated chronic left heart failure: a rare case report

**DOI:** 10.1097/MS9.0000000000003756

**Published:** 2025-09-01

**Authors:** Mohanad Jaber, Majed Dwaik, Amjed Abedalnabi, Nidal Jebrini, Husein Sarahneh, Nagham A Shalaldeh, Sadil Majed Shalaldeh, Abdalhay A. J. Asafra

**Affiliations:** a Faculty of Medicine, Palestine Polytechnic University, Hebron, Palestine; b Dean’s Office, Faculty of Medicine, Palestine Polytechnic University, Hebron, Palestine; c Head of IM department Faculty of Medicine, Palestine Polytechnic University, Hebron, Palestine; d Hemato-oncology Department, Palestine Polytechnic University, Hebron, Palestine; e Radiology Department, Al-Ahli Hospital, Hebron, Palestine

**Keywords:** acute abdominal pain, case report, heart failure with reduced ejection fraction (HFrEF), left-sided heart failure, omental infarction

## Abstract

**Introduction and importance:**

Omental infarction (OI) is a rare cause of acute abdominal pain, frequently misidentified as appendicitis or diverticulitis. It generally originates from vascular compromise, torsion, or thrombosis and is infrequently associated with left-sided heart failure. This appears to be the inaugural documented instance of OI linked to left-sided heart failure.

**Case presentation:**

A 43-year-old man with a history of coronary artery disease and heart failure with reduced ejection fraction (35%) presented with right lower quadrant abdominal pain initially localized to the periumbilical region. CT imaging demonstrated distinctive fat stranding and haziness indicative of OI. The patient exhibited hemodynamic stability and was treated conservatively with symptomatic and supportive care, including medications for heart failure. He exhibited clinical improvement and was discharged in satisfactory condition.

**Clinical discussion:**

This case underscores the possible association between heart failure and OI. Hemodynamic instability and prothrombotic conditions in chronic heart failure may lead to diminished omental perfusion. CT imaging is essential for diagnosis, allowing for differentiation from surgical emergencies like appendicitis. Conservative treatment is efficacious in the majority of instances and may avert superfluous surgical procedures.

**Conclusion:**

Clinicians must include OI in the differential diagnosis of acute abdominal pain, even in patients lacking conventional risk factors. In individuals with cardiac comorbidities, identifying this uncommon association may facilitate suitable nonoperative management and diminish morbidity.

## Introduction

Omental infarction (OI) is an uncommon etiology of acute abdominal pain, frequently misidentified as appendicitis or cholecystitis. It usually arises from vascular compromise, such as torsion or thrombosis, and is more frequently identified through contrast-enhanced CT, which reveals fat stranding and fatty masses^[1,2]^. Left-sided heart failure (HFrEF) may also induce organ ischemia via hemodynamic instability and venous congestion, which diminishes perfusion to the omentum^[3,4]^. Timely diagnosis is vital to distinguish omental infarction from other abdominal disorders, and although the majority of cases resolve with conservative treatment, precise imaging is imperative to prevent unwarranted surgical interventions^[5]^. This report has been done based on SCARE 2025 Guidelines^[6]^.

**HIGHLIGHTS**

- This case report discusses a rare occurrence where left-sided heart failure manifested as acute abdominal pain due to omental infarction.
- The condition was diagnosed using contrast-enhanced CT imaging, which revealed characteristic signs of fat stranding and haziness.
- The patient was treated conservatively with heart failure medications and antibiotics.
- Remarkably, no surgical intervention was required.
- The case highlights the importance of considering omental infarction as a potential complication in patients with heart failure and abdominal pain.
- It also emphasizes that conservative management can be effective in some cases.

## Case presentation

A 43-year-old man was referred to our hospital from an outpatient clinic to rule out acute appendicitis.

The patient chiefly complained of shortness of breath which started 10 hours before admission, accompanied by continuous, gradually progressive pain in the periumbilical region. The pain initially radiated before localizing to the right lower quadrant (RLQ) of the abdomen. He denied nausea, vomiting, chills, fever, itching, melena, any change in bowel habits, or in stool or urine color.

The patient’s past medical history was significant for coronary artery disease and HFrEF (50%). Five years ago, he underwent cardiac catheterization with stent placement. His medication regimen included: Crestor (rosuvastatin) 20 mg once daily, Hypocor (bisoprolol) 5 mg once daily, Aspirin 100 mg once daily, Plavix (clopidogrel) 75 mg once daily, and Pantover (pantoprazole) 40 mg once daily.

On physical examination, he appeared ill and uncomfortable. He was febrile (38.2°C), tachycardic (105 beats/min), and normotensive (107/61 mmHg). His oxygen saturation was 91% on room air and his body mass index was 28 kg/m^2^. On abdominal palpation, the abdomen was soft, non-distended, with RLQ tenderness, rebound tenderness, and a positive Rovsing sign. No mass or organomegaly was palpated. Cardiopulmonary examination was unremarkable.

Laboratory examination showed leukocytosis (white blood cells: 13 800/μL) with a left shift (71.7% neutrophils). Biochemical investigations, including renal, hepatic, and coagulation profiles, were within the normal reference ranges.

A timeline of symptom onset, evaluation, and treatment is summarized as follows: symptoms began 10 hours prior to admission; CT imaging and echocardiography were performed on the day of admission; conservative management was initiated the same day; the patient was discharged within 24 hours.

**Figure 1.**
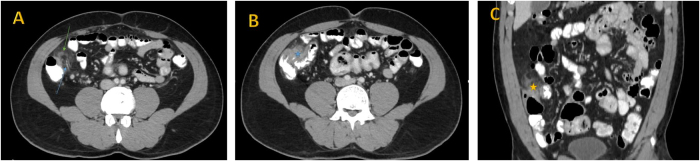
Selected axial (A + B) and coronal (C) abdomen CT scan with intravenous contrast images were done for our patient . They are showing a focal and oval shaped area of omental haziness is located along the anteromedial aspect of the ascending colon near the ileocecal valve (green arrow in A image and yellow star in C image), measuring 4.9 × 3.3 × 2.7 cm in craniocaudal, anteroposterior and transverse diameters respectively . Its associated with wall thickening of the adjacent part of ileocecal valve (blue star in B image). Note the small omental vessels entering the center of the haziness area (blue arrow in A image). These finding are suggestive of omental infarction.

A contrast-enhanced abdominal CT scan (IV and oral contrast) revealed a focal area of fat haziness and stranding anterior to the cecum and the terminal ileum associated with focal circumferential wall thickening of the affected part of the terminal ileum and cecum, and to a lesser extent there was mild mural thickening of the transverse colon. A few prominent mesenteric lymph nodes were noted at the right iliac fossa as illustrated in Fig. 1. The appendix was of normal diameter without significant peri-appendiceal fat stranding. The radiological findings were consistent with acute OI or, less likely, epiploic appendagitis. A transthoracic echocardiogram revealed a reduced EF of 35%, compared to the patient’s last documented EF of 50% in 2021.

Differential diagnoses included acute appendicitis, epiploic appendagitis, and cecal diverticulitis; these were excluded based on clinical, laboratory, and imaging findings.

Upon general surgery team consultation, conservative management was recommended. The patient was admitted for observation and managed conservatively including intravenous fluid, Pramin (metoclopramide), empiric antibiotics (Rocephin and Flagyl), and a proton pump inhibitor as prophylaxis. Entresto (sacubitril/valsartan) 100 mg in the morning and 50 mg in the evening was initiated, and home medication was continued. Six hours later, he exhibited symptomatic improvement but was discharged against medical advice.

Upon discharge, the patient was prescribed the following: Pramin 10 mg three times daily as needed, Scobutyl (hyoscine butylbromide) ampoule as needed, Ciprofloxacin 500 mg twice daily for 5 days, Metronidazole (Flagyl) 500 mg three times daily for 5 days, and Colchicine 0.5 mg twice daily. Antibiotic therapy was given due to leukocytosis, fever, and radiological evidence of inflammation.

## Discussion

OI is an uncommon but important differential diagnosis in patients presenting with acute abdominal pain, particularly when located in the right lower quadrant^[1]^. OI has a very low incidence of approximately 0.3% of all patients presenting with acute abdomen to the emergency departments and is found in 0.1% of laparotomies for acute abdominal pain^[1]^. According to the literature, this is the first reported case of omental infarction associated with left-sided heart failure.

Idiopathic (primary) OI occurs when the underlying cause remains unclear. Several theories suggest possible mechanisms, including congenital vascular abnormalities that predispose to thrombosis, excessive fat deposition in the omentum increasing gravitational stress, and an imbalance between omental tissue growth and its vascular supply, leading to ischemia and necrosis^[3]^. Secondary causes include omental torsion, trauma, inflammatory processes, or neoplastic diseases^[5]^. The omental blood supply is composed of numerous small vessels with limited collateral circulation, making it susceptible to ischemic injury in cases of vascular compromise^[5]^. While arterial occlusions are rare, venous infarction is more commonly observed, often linked to thrombosis, congestion, or external compression^[5]^.

Epidemiologically, OI is more frequently observed in men aged between 30 and 40, with a male-to-female ratio of approximately 2:1^[2]^.

Several risk factors have been associated with OI, including obesity, recent abdominal surgery, trauma, hypercoagulable states, and sudden body movements that cause omental torsion^[2]^. Although our patient had no history of recent surgery or direct trauma, his heart failure with reduced ejection fraction (HFrEF) increased his risk of infarction^[2]^.

In this case, the patient, a 43-year-old man, presented with progressive RLQ pain, initially periumbilical. His symptoms were not accompanied by nausea, vomiting, changes in bowel habits, fever, melena, jaundice, itching, or systemic signs of infection. Given the clinical presentation and imaging findings, a diagnosis of OI/epiploic appendagitis was made.

**Figure 2.**
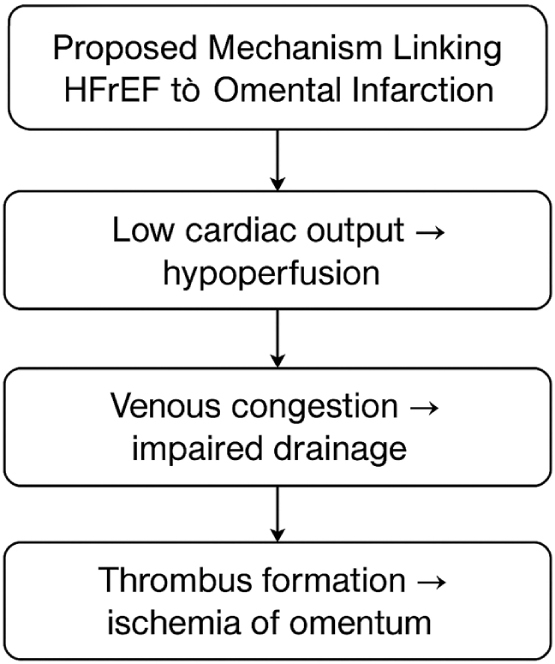
Proposed mechanism of omental infarction in HFrEF.

In our case, the patient had a known history of chronic HFrEF, with recent imaging indicating a decline in EF from 50% to 35%, consistent with a subacute worsening rather than an acute decompensation. Chronic heart failure is associated with systemic venous congestion and low perfusion states, which may contribute to microvascular ischemia, including rare events such as OI (see Fig. 2).

CT imaging confirmed fat stranding and haziness anterior to the cecum and terminal ileum, with no evidence of peri-appendiceal fat stranding or fecalith, effectively ruling out acute appendicitis. These radiological findings were consistent with OI rather than other common causes of RLQ pain^[4]^. Given its rarity and nonspecific symptoms, more common causes of acute abdomen – such as appendicitis, cholecystitis, and diverticulitis – must be excluded^[7]^.

OI can be differentiated from acute appendicitis and cholecystitis on a CT scan by identifying a normal, non-inflamed appendix and gallbladder^[8]^. Epiploic appendagitis, however, presents a greater diagnostic challenge, as both conditions exhibit a fatty mass on imaging. The key distinguishing feature is lesion size; in epiploic appendagitis, lesions are typically smaller than 5 cm, whereas OI lesions tend to be larger^[9]^. Refer to Table 1 to distinguish between these two similar diagnoses. Cecal diverticulitis, perforated duodenal ulcer, abdominal wall hematoma, and intestinal obstruction are other differential diagnoses of OI^[10]^. Diverticulitis can be ruled out based on the absence of inflamed diverticula, significant bowel wall thickening, or paracolic abscesses^[11]^. However, some reports suggest mild bowel wall thickening in OI due to localized inflammatory spread^[9]^. No intra-abdominal free air was observed, effectively ruling out abdominal perforation^[9]^. Furthermore, various benign and malignant neoplasms – such as lipoma, angiomyolipoma, teratoma, mesenteric lipodystrophy, peritoneal pseudomyxoma, liposarcoma, and peritoneal mesothelioma – may present as fatty masses on imaging, but their distinct clinical contexts usually help avoid diagnostic confusion^[9]^.

**Table 1:** Comparison between omental infarction and epiploic appendagitis

Feature	Omental infarction	Epiploic appendagitis
Typical size	>5 cm	<5 cm
Location	Greater omentum (commonly right side of abdomen)	Along the colon, especially sigmoid and descending
Onset of pain	Gradual and dull	Sudden and sharp
Vascular supply	Omental vessels (from gastroepiploic arteries)	Epiploic vessels (branches of colonic vessels)
CT findings	Fat stranding and focal fatty mass near cecum	Small, oval, fat-density lesion with central dot sign
Associated findings	Adjacent bowel wall thickening may occur	No bowel wall thickening
Systemic signs	May have mild fever and leukocytosis	Usually afebrile, normal white blood cells
Treatment	Often conservative (rarely surgical if complications)	Conservative (self-limiting)
Prognosis	Excellent with conservative management	Excellent with conservative management

OI occurs due to impaired blood flow to the omentum, often resulting from thrombosis of small omental veins, leading to hemorrhagic necrosis^[12]^. One major cause is right-sided heart failure, which leads to severe venous congestion, ultimately reducing perfusion to the omentum^[12]^.

Patients typically present with RLQ pain^[12]^. Cases of OI associated with both right-sided and left-sided heart failure share nonspecific symptoms, making diagnosis challenging. They share common risk factors such as obesity, hypercoagulability, venous congestion, abdominal trauma, and prior surgeries^[12]^. Diagnosis is typically made using CT imaging, which reveals venous congestion and focal fat infiltration, confirming OI^[12]^.

In these cases, surgical resection of the infarcted omentum was performed, but surgical management increases the risk of complications such as peritonitis, bowel obstruction, and adhesions^[12]^. In contrast, our patient was managed conservatively, with successful resolution of symptoms and no complications.

The mainstay of treatment for OI is conservative management, including analgesia, anti-inflammatory medications, and supportive care. Studies indicate that 95.5% of patients are successfully managed nonoperatively without complications^[12]^. Surgical intervention is typically reserved for cases complicated by necrosis, abscess formation, or diagnostic uncertainty^[12]^. Potential complications of OI include abscess formation, intra-abdominal adhesions, bowel obstruction, and, in rare cases, perforation of the infarcted tissue. Surgical intervention may be necessary to prevent such complications in select cases^[4]^.

The patient was managed conservatively with symptomatic treatment, antibiotics, and medications for his underlying cardiovascular disease. He was prescribed intravenous fluid, Pramin (metoclopramide), empiric antibiotics (Rocephin and Flagyl), and a proton pump inhibitor as a prophylaxis. Home medications were continued, including Aspirin (81 mg once daily), Clopidogrel (75 mg once daily), Rosuvastatin (20 mg once daily), Bisoprolol (2.5 mg once daily), and Gabapentin (dose unclear, once daily). Upon discharge, Pantoprazole 40 mg once daily for gastric protection, Pramin 10 mg up to three times daily as needed, and Scobutyl (Hyoscine) injection 1 ampoule up to three times daily as needed for nausea and abdominal cramps were prescribed. Empirical antibiotic therapy included Ciprofloxacin 500 mg twice daily for 5 days and Metronidazole (Flagyl) 500 mg three times daily for 5 days to reduce the risk of secondary infection. Additionally, he was on Crestor (Rosuvastatin) 20 mg once daily for lipid management, Hypocor (Bisoprolol) 5 mg once daily as a beta-blocker, Forxiga (Dapagliflozin) 10 mg once daily as an SGLT2 inhibitor, Entresto (Sacubitril/Valsartan) 100 mg in the morning and 50 mg in the evening, Aspirin (ASA) 100 mg once daily, Plavix (Clopidogrel) 75 mg once daily as antiplatelet therapy, and Colchicine 0.5 mg twice daily for inflammatory modulation. These medications may have influenced the course of the infarction. Antiplatelet agents such as aspirin and Clopidogrel may impair localized thrombus formation, potentially affecting the extent of ischemia. Dapagliflozin, through volume contraction, might contribute to visceral hypoperfusion, whereas Sacubitril/Valsartan, via vasodilatory and anti-inflammatory effects, could theoretically mitigate ischemic injury. Colchicine, with its potent anti-inflammatory properties, may have reduced the inflammatory response, lowering the risk of complications like abscess formation or adhesions. Bisoprolol, as a beta-blocker, might contribute to mesenteric hypoperfusion due to its hemodynamic effects. Despite these theoretical concerns, the patient recovered well without any complications, further supporting the effectiveness and safety of conservative management in OI, even in patients with chronic heart failure. His condition remained stable throughout hospitalization and upon discharge. Table 2 illustrates the theoretical impact of several heart failure medications on OI.

**Table 2:** Theoretical impact of medications on OI

Medication	Class/Action	Potential theoretical effect on omental infarction
Aspirin (ASA)	Antiplatelet	↓ Platelet aggregation → may impair local thrombus formation; possibly reducing protective clot formation
Clopidogrel (Plavix)	Antiplatelet (P2Y12 inhibitor)	Similar to aspirin; ↑ bleeding risk; ↓ thrombus formation may exacerbate infarction zone
Rosuvastatin (Crestor)	Statin (Lipid-lowering, anti-inflammatory)	May provide vascular protective and anti-inflammatory effects → potentially beneficial
Bisoprolol (Hypocor)	Beta-blocker	↓ Cardiac output → may contribute to mesenteric/omental hypoperfusion
Sacubitril/Valsartan (Entresto)	ARNI (vasodilatory, natriuretic)	Vasodilation and anti-inflammatory effects → may improve perfusion and reduce ischemic injury
Dapagliflozin (Forxiga)	SGLT2 Inhibitor	Osmotic diuresis → intravascular volume contraction → ↓ perfusion to abdominal organs
Colchicine	Anti-inflammatory	↓ Neutrophil activity and inflammatory response → may reduce complications (e.g., abscess, adhesions)
Pantoprazole (Pantover)	Proton pump inhibitor	No direct effect on infarction; used for gastric protection due to NSAIDs or antibiotics
Metoclopramide (Pramin)	Antiemetic/Prokinetic	Symptomatic relief of nausea; no direct influence on infarction
Hyoscine (Scobutyl)	Antispasmodic	Relieves abdominal cramping; no direct effect on the underlying infarct
Gabapentin	Neuropathic pain modulator	Unrelated to infarction; used for neuropathic symptoms or chronic pain
Ciprofloxacin	Broad-spectrum antibiotic	Prevents or treats secondary infection in infarcted tissue
Metronidazole (Flagyl)	Anaerobic antibiotic	Similar to ciprofloxacin; adds anaerobic coverage to prevent superinfection

## Conclusion

OI is a rare and frequently disregarded etiology of acute abdominal pain, especially in individuals lacking traditional risk factors. This case distinctly emphasizes the correlation between left-sided heart failure and OI, a connection not previously documented in the literature. Early identification via imaging and the exclusion of surgical emergencies can enable conservative treatment, preventing unwarranted procedures. Clinicians must uphold a heightened level of suspicion for OI in atypical cases, particularly in patients with cardiovascular disease.

## Data Availability

The corresponding author has a full access to all data and takes responsibility for its integrity and accuracy.

## References

[1] Wiesner W Mortelé KJ Glickman JN. Primary omental infarct: CT findings with pathologic correlation. Clin Imaging 2003;27:31–33.12504318

[2] Ciarrocchi A Amadori M Guidi E. Omental infarction: a rare cause of acute abdomen. Int J Surg Case Rep 2023;107:108268.37187113

[3] Leitner MJ Jordan CG Spinner MH. Torsion of the omentum: review of 98 cases. Surgery 1952;32:1031–36.

[4] Rioux M Langis P. Primary epiploic appendagitis: clinical, US, and CT findings in 14 cases. Radiology 1994;191:523–26. 8153333 10.1148/radiology.191.2.8153333

[5] Wiesner W Mortelé KJ Glickman JN. Radiologic-pathologic correlation of omental infarction: imaging findings and clinical implications. Clin Imaging 2003;27:31–33.12504318

[6] Sohrabi C Mathew G Maria N. The SCARE 2023 guideline: updating consensus Surgical CAse REport (SCARE) guidelines. Int J Surg Lond Engl 2023;109:1136. 10.1097/JS9.0000000000000373 PMC10389401 37013953

[7] Singh AK Gervais DA Hahn PF. Omental infarction: CT imaging features. Eur J Radiol 2002;43:114–17.

[8] Baloch NU Qazi I Zaman M. Primary omental infarction masquerading as appendicitis: a rare case from Pakistan. Cureus 2021;13:e13439.33763322

[9] Cianci R Filippone A Basilico R. Idiopathic segmental infarction of the greater omentum diagnosed by unenhanced multidetector-row CT and treated successfully by laparoscopy. Emerg Radiol 2007;15:51–56. 17610001 10.1007/s10140-007-0631-z

[10] Goti F Hollmann R Stieger R. Primary torsion of the omentum: a rare cause of acute abdomen. Eur J Surg 1998;164:973–76.

[11] Jeffrey RB Federle MP Crass RA. CT findings in patients with pericolonic inflammatory disease: diverticulitis and epiploic appendagitis. AJR Am J Roentgenol 2002;178:815–19.

[12] Andreuccetti J Ceribelli C Manto O. Omental infarction: a cause of acute abdomen not to be underestimated. Emerg Radiol 2003;10:28–30.

